# Tensile Strength Improvements of Ramie Fiber Threads through Combination of Citric Acid and Sodium Hypophosphite Cross-Linking

**DOI:** 10.3390/ma16134758

**Published:** 2023-06-30

**Authors:** Asri Peni Wulandari, Vira Putri Dinda Awis, Ruly Budiono, Joko Kusmoro, Sidiq Syamsul Hidayat, Nanang Masruchin, Muhammad Adly Rahandi Lubis, Widya Fatriasari, Ulyaa Rachmawati

**Affiliations:** 1 Department of Biology, Faculty of Mathematics and Natural Science, Universitas Padjadjaran, Sumedang 45363, Indonesia; vira18006@mail.unpad.ac.id (V.P.D.A.); ruly.budiono@unpad.ac.id (R.B.); joko.kusmoro@unpad.ac.id (J.K.); ulyaa19001@mail.unpad.ac.id (U.R.); 2 Center for Study of Bioprospection of Natural Fiber and Bioresources, Faculty of Mathematics and Natural Science, Universitas Padjadjaran, Bandung 40132, Indonesia; 3 Program for Study of Telecommunications Engineering, Electrical Engineering Major, Faculty of Engineering, Politeknik Negeri Semarang, Semarang 50275, Indonesia; sidiqsh@polines.ac.id; 4 Research Center for Biomass and Bioproduct, National Research and Innovation Agency, Bogor 16911, Indonesia; nana021@brin.go.id (N.M.); marl@biomaterial.lipi.go.id (M.A.R.L.); widy003@brin.go.id (W.F.)

**Keywords:** *Boehmeria nivea*, cross linking, natural fiber, tensile strength

## Abstract

Ramie (*Boehmeria nivea*) is believed to be one of the strongest natural fibers, but it still remains behind synthetic materials in terms of tensile strength. In this study, ramie materials were prepared to evaluate the modification crosslinking effect of natural fiber. The aim is to optimize various concentrations of citric acid (CA) crosslinking by adding Sodium hypophosphite (NaPO2H2), which is activated at different temperatures, to obtain the highest tensile mechanical strength. This crosslinking effect has been confirmed by FTIR to show the esterification process in the molecular structure of cellulose. The changes in the character of the fiber surface were analyzed by SEM. The tensile strength increased from 62.33 MPa for 0% CA to 124–172.86 MPa for decorticated fiber with a CA concentration of 0.75–1.875% (*w*/*w*). A significant increase in tensile strength was observed more than 19 times when CA/SHP 1% was treated at an activation temperature of 110 °C with a superior tensile strength of 1290.63. The fiber crosslinked with CA/SHP should be recommended for application of Natural Fiber Reinforced Polymer Composite (NFRPC), which has the potential to use in functional textile and industrial sector automotive or construction.

## 1. Introduction

The toughness of a fiber’s mechanical properties can be determined by its technical parameters, such as strength, thermal resistance, or other attributes. High air diffusivity and hydrophilicity in natural fibers cause a substantial amount of water in plant fibers, which can increase biomass weight, promote weathering, and weaken fibers. Due to their inferior mechanical qualities compared to synthetic fibers, natural fibers are still challenging to use today.

Ramie fiber, which contains 80–85% cellulose, is mostly utilized as a raw material for textiles [1]. Ramie also has superiority in terms of productivity, with 200–237.5% higher productivity yield (2–2.7 tonnes per hectare per year) [2] compared to cotton, with only a yield of 0.8 tonnes per hectare per year (global average) [3], thus making it way more cost and land efficient than cotton as natural fibers.

It stands out among other natural fibers for its exceptional mechanical characteristics and highest thermal conductivity [4]. Ramie can withstand tensile loads three times as effectively as cotton fiber, but it still remains behind synthetic materials in terms of tensile strength [5].

Many methods have been studied in recent years to enhance some of the features of ramie by changing the molecular structure and surface characteristics of the fiber and the cellular processes necessary for polymer impregnation, such as by treating them in alkaline, enzymatic, or crosslinked forms. Improvements in mechanical, thermal, and adhesion have been made using anionic polyamide-6 (APA-6) [6], polylactic acid (PLA) combined with isophorone diisocyanate (IPDI) compatibilizer [7], and diammonium phosphate (DAP) as a pretreatment for PLA/ramie applications [8]. Polyurethane non-isocyanate resin (NIPU) is made from tannins [9], while another type of resin called polyurethane resin (LPU) is made from low-viscosity lignin [10]. There is a way to remove hair from ramie fabrics using a special technology that includes a mixture of NaOH and urea, making the fabric stronger and less likely to absorb water [11]. The fabric can absorb dyes better and maintain its color longer by adding a certain type of polymer with an aliphatic mechanism, such as aliphatic amino-terminating polymer (HBP-NH_2_) and amino-terminating hyperbranched polymer (at-HBP) [12,13].

The chemical procedure known as crosslinking is commonly employed within the cellulose textile sector to address the issues pertaining to mechanical properties and dimensional instability [14]. Some of the new studies related to the use of environmentally friendly natural crosslinking materials are from the Polycarboxyl Acid (PCA) group, which includes carboxylic acid groups such as Maleic acid (MA), Malic acid (MLA), Succinic acid (SUA), Citric acid (CA), and 1,2,3,4-butane tetracarboxylic acid (BTCA) were reported to be potentially reactive with the cell wall in the fiber surface modification process because they have -COOH groups that can crosslink with cellulose polymers in the cell wall [15]. In addition, the application of a crosslinking agent from CA can use three carboxylic groups per molecule to facilitate the optimization of the synthesis process with several other advantages, such as readily accessible, cost-effective, and more environmentally sustainable [16,17].

Several kinds of fiber have been shown to have CA crosslinking treatment, including improvement of mechanical properties and the thermal stability of bamboo [18]; enhanced in cotton fabrics treated with CA/chitosan and graphene oxide improved color fastness, crease resistance, and antibacterial properties that were effective against Gram-positive and Gram-negative bacteria [19], and mechanical properties (of flexural strength and hardness) as well as the thermal stability of kenaf [20]. CA-cellulose *Ceiba pentandra* has no cytotoxic effects on human skin fibroblasts. It is appropriately used in the pharmaceutical sector as a manufacturing material for drug release systems [21].

The majority of recent studies on fiber modification with CA crosslinking improved the effectiveness by including other chemicals, such as: bleaching with hydrogen peroxide may reduce the yellowing effect and maintain the anti-wrinkle effect of citric acid treated fabrics under proper alkaline conditions, short times, and high temperatures [17].

Several kinds of catalysts, including monosodium phosphate, sodium carbonate, and N-heterocyclic, can accelerate the esterification reaction during the crosslinking process, although their use can be rather costly [22]. As a catalyst for the crosslinking process employing the citric acid crosslinker, SHP has been found to be the most effective [23]. Application in a wood combination of CA/SHP reduced the mobility of the carboxyl group (-COOH) from CA so as to minimize cellulose degradation [24].

In this research, to the best of our knowledge, there has been no report on using ramie cellulose fiber to be crosslinked with CA to improve tensile properties. The goal is to optimize the composition of CA as a crosslinker with the addition of SHP catalyst examined on decorticated ramie fiber at a specific temperature level in order to obtain the highest mechanical strength value. The best CA/SHP composition was tested on degummed-fibers and ramie yarn to see how the strength properties changed.

## 2. Materials and Methods

### 2.1. Materials

**Figure 1:**
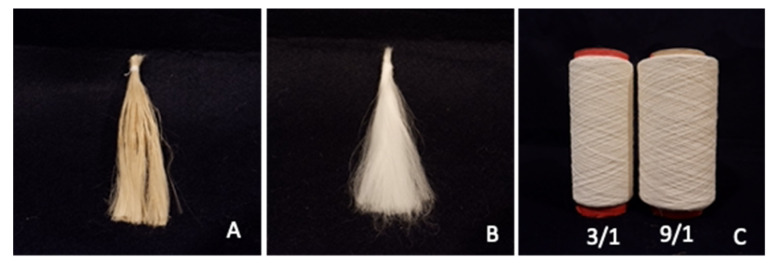
Ramie material for evaluation of CA/SHP crosslinking: (**A**) decorticated-fiber, (**B**) degummed-fiber, and (**C**) ramie yarn: code Yarn A_RmRy3/1; and Yarn type_RmRy9/1.

The ramie samples (*Boehmeria nivea* L. Gaud) that were used for the material in the crosslinking test are decorticated fiber (cellulose content 80–85%), chemically degummed-fiber as fine fiber with a diameter of 0.2–0.13 mm with code product: Rfiber_CD/hthp_P01 and two types of product ramie yarn with code Yarn A_RmRy3/1; and Yarn type_RmRy9/1. Additionally, chemicals citric acid and sodium hydrophosphite were supplied from PT RAJANTARA, Indonesia (Figure 1). The method of Wulandari (2021) [4] was used to produce bio-degummed-fiber, and the preparation of the starter was adopted from Wulandari, 2016 [25].

### 2.2. Preparation of CA-Treated Specimens

The crosslinking process refers to the method carried out by Hassan et al., 2020 [26] with some modifications. The decorticated fiber 2.5% (*w*/*v*) was submerged in water. The fibers were then treated with variations of citric acid (0.5%, 0.75%, 1.25%, 1.875%, and 2.5%). Sample decorticated fiber without immersion treatment will act as a control. The fiber specimens were immersed in CA solution for 3 h.

All specimens were rinsed off with deionized water to remove any remaining CA. Furthermore, the fibers were dried for 8–10 h at 90–110 °C in a drying cabinet (Memert, Schwabach, Germany, type UN30 230 V-50/60 Hz DIN12880-2007-Kl). The resulting samples were ready to be analyzed for tensile strength. The storage conditions for samples prior to testing were at room temperature between 25–27 °C.

### 2.3. Optimization of Catalyst SHP and Activation Temperature

One of the specimens with maximum tensile strength was used to estimate the ideal CA concentration. The chosen formula was then investigated for the effect of employing SHP as a catalyst with varying concentrations of 1–2% (*w*/*w*) and activated at various temperatures 90 °C, 100 °C, and 110 °C.

### 2.4. Mechanical Tensile Strength Analysis

Testing the resistance characteristics of the mechanical properties of the fiber is needed to measure the tensile strength, which refers to Pua et al. (2013) [20].

**Figure 2:**
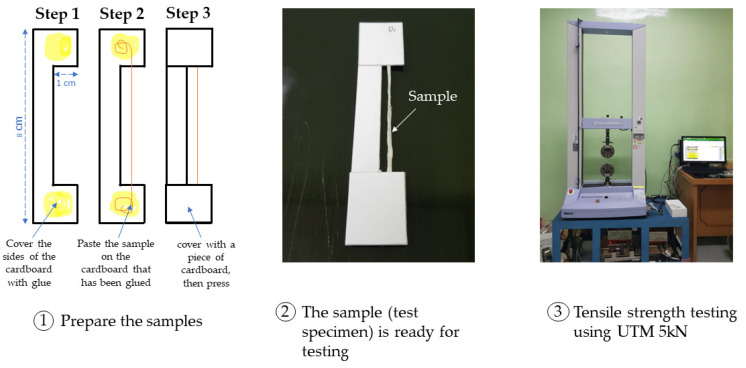
Instruments UTM for Tensile Analysis.

The analysis procedure determined by American Standard Testing Materials (ASTM) D3379 [27] by using an instrument, the Shimadzu Universal Testing Machine (UTM) AGS-X type X series 5 kN from BRIN Laboratory of Advanced Characterization, in Cibinong, West Java, Indonesia (Figure 2). The fiber specimens to be tested were randomly cut to a length of 15–20 cm, and five times the fiber was cut for each condition. All samples were conditioned for at least 48 h prior to testing at 25–27 °C.

### 2.5. Fourier Transform Infra-Red (FTIR) Analysis

FTIR analysis was carried out to determine the effect of the test treatment on the functional group content of ramie fiber. Tensile strength for both CA only and CA + SHP are validated by test standard BS EN ISO 527-2:1996 [28]. Analysis was performed with PerkinElmer FTIR (Model: System 2000, PerkinElmer Corporation, Waltham, MA, USA) using the ATR (Attenuated Total Reflectance) method with Spectrum Two Specs, resolution 4 cm^−1^, and 16 scans. This FTIR analysis will produce data in the form of absorbance or transmittance graphs. FTIR analysis was then carried out quantitatively by paying attention to the shape of the spectra at specific peaks and the intensity of the FTIR graph. The test was carried out in the range of wave numbers 400–4000 cm^−1^. The resolution was settled at 2 cm^−1^ to observe absorption peaks of the OH (hydroxyl) group at wave numbers 3000–3650 cm^−1^ and -COOR′ (ester) absorption at wave numbers 1710–1780 cm^−1^.

### 2.6. Micrograph Surface Structure Analysis

The surfaces of the structural fibers were observed by Scanning electron microscope (SEM) imaged with FE-SEM Thermo Scientific Quattro S completed with EDS Detector, WetSTEM, Heating Stage, and Tensile Stage. The samples were coated with gold using a Cressington 208HR High-Resolution sputter coater (Cressington Scientific Instruments, Watford, UK). Magnification of 100–10,000 times until the surface object was visible.

## 3. Results

### 3.1. Effect of Citric Acid (CA) on Tensile Strength

**Figure 3:**
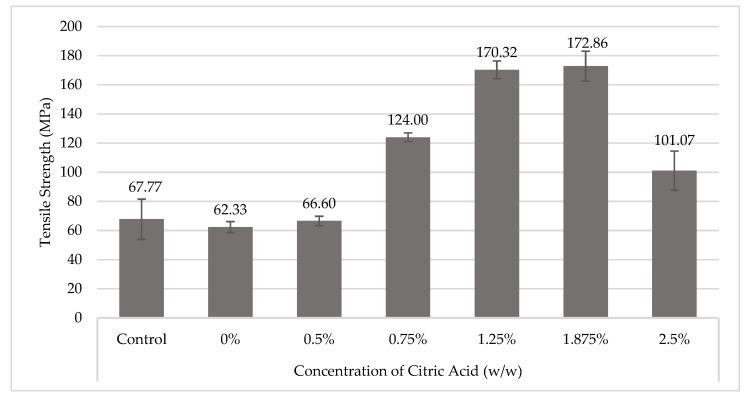
Tensile strength of Crosslinked-CA to ramie fiber.

Tensile strength tests were carried out for crosslinked CA fibers. The changes of the tensile with various concentrations of CA are shown in Figure 3. In ramie fiber, CA crosslinking has a positive effect on mechanical properties. The control showed a tensile strength of 67.77 MPa, which decreased slightly by about 4% when immersed in water (CA 0%). Citric acid is available in the monohydrate form (contains one molecule of water), so it needs to be activated first in the form of anhydrous/cyclic (water-free) anhydride in order to bind to the hydroxyl groups on the fiber surface. Therefore, with a weak organic acid group, it appears to have a limited ability to dissociate with OH- ions at concentrations below 0.5%.

The tensile strength of the fiber will increase with the addition of CA at a gradually increasing concentration. Adding CA 0.75–1.875% (*w*/*w*) can show a significant increase in tensile strength up to 124 MPa–172.86 MPa or around 83–155% compared to the control fiber. The concentration of carboxylate ion (-COOH) saturation due to the addition of CA at 2.5% will limit the mobility of CA and could be acid-catalyzed hydrolytic breakdown of cellulose would reduce the crosslinking efficiency of the fiber, resulting in lower tensile strength.

### 3.2. Crosslinking Reaction with Addition of Sodium Hypophosphite (SHP) and Temperature

**Figure 4:**
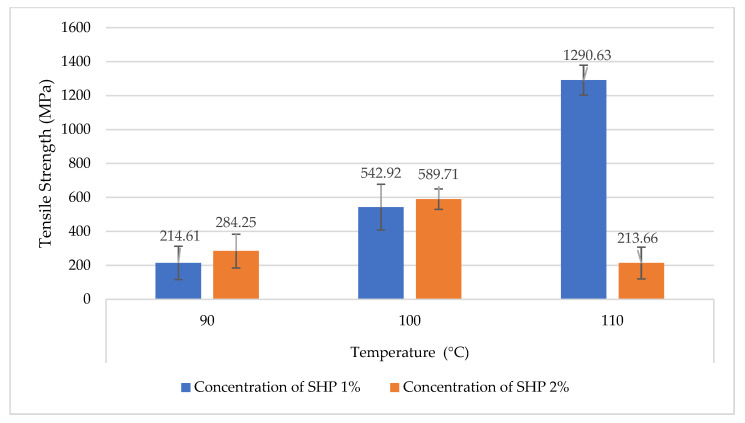
Tensile strength of crosslinked-CA to ramie that activated with SHP at different temperatures.

The increase in mechanical performance, especially the tensile strength of crosslinked CA in ramie fiber, seems to be dependent on the curing temperature (Figure 4). Citric acid applications can react to ramie fiber in the absence of the catalyst SHP. Therefore, the research was continued by applying the optimal concentration of 1.875% CA with 1% and 2% SHP, accelerated at various activation temperatures of 90 °C, 100 °C, and 110 °C. The result of the change of crosslinking on the tensile strength is shown in Figure 3. When observed with activation temperature at 90 °C, the usage of 1% and 2% SHP showed a tendency to increase to 214.61 MPa (216%) and 284.25 MPa (319%), respectively. Furthermore, the crosslinked CA to fiber gave effects at 100 °C at 1% and 2% of SHP were 542.92 MPa (701%) and 589.71 MPa (770%), respectively. Surprisingly, the strength of the crosslinked-CA with ramie fiber increased more than 19 times when the activation temperature was raised by 110 °C compared to the control (67.77 MPa), reaching a maximum tensile strength of 1290.63 MPa (1804%). However, these results were not applied when 2% SHP was used, which caused a decline in fiber strength.

The increase in the fiber strength during the crosslinking process with CA/SHP can occur if the CA structure forms a monohydrate from one water molecule to bind the hydroxyl group on the fiber surface, for that CA must first be activated in the form of anhydrous/cyclic anhydride, which is free of water. In this experiment, SHP played an effective role in the crosslinking reaction with CA at 110 °C. As a catalyst, SHP enhanced the decomposition of CA under thermal conditions for esterification in fiber and reduced the unsaturated intermediates to methylsuccinic acid [29].

Phosphate binding takes place in the fiber by the H–P–H reaction of SHP with the C=C from CA, which causes phosphate anions to diffuse deeper into the fiber polymer, causing more crosslinks to form [30]. Therefore, it is the potential to promote the formation of anhydrous cyclic intermediate molecules to accelerate the reaction between the intermediate and the hydroxyl groups of cellulose [31]. At higher temperatures, this condition might also reduce the effectiveness of crosslinking in cellulose.

### 3.3. FTIR Spectra

**Figure 5:**
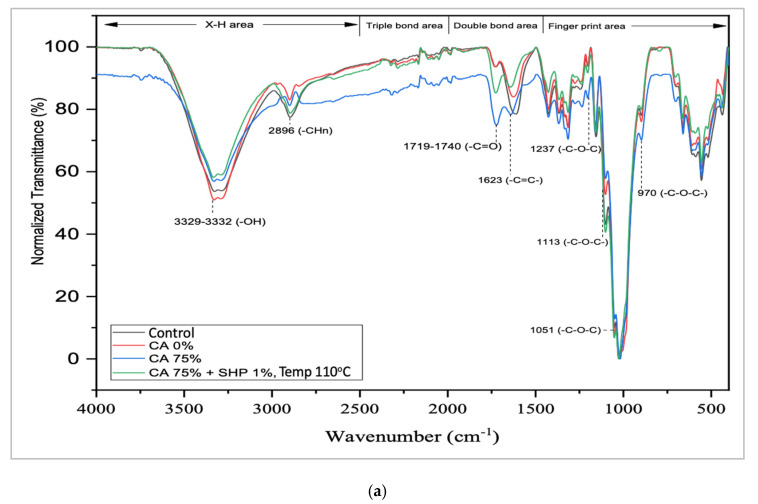

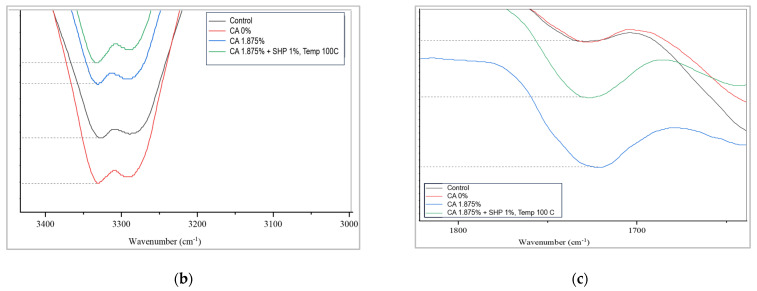
FTIR spectra of ramie fiber crosslinked with CA 1.875% and CA 1.875% + SHP, Temp. 110 °C. (**a**) Typical spectra (**b**) expand region 3400–3000 (cm^−1^) (**c**) expand region 1800–1750 (cm^−1^).

Changes in functional groups of ramie fiber in the CA/SHP crosslinking were shown in Figure 5. CA has a tricarboxylic group (-COOH) that can create group ester bridges (-COOR′) when it is crosslinked with hydroxyl groups (-OH) on the surface of the fiber. The properties of the -OH hydroxyl group in cellulose, including hemicellulose and lignin, appear in the band 3329–3332 cm^−1^.

The peak’s absorption is increased by the residual water in the fiber, demonstrating the presence of -OH groups. The treatment in this study led to a decrease in the intensity of the hydroxyl group with alterations because of the reaction between the hydroxyl groups of cellulose and the positive carbon of the CA carboxyl group, which combined to generate the ester group.

The C-H unsaturated stretching mode of CA can cause a change in the carboxyl function group (C=O), indicated at wave number 1719 cm^−1^. When heated, the reaction starts with the hydration of CA to form a cyclic anhydride followed by esterification with an OH-group of cellulose [32,33]. A peak of 1.435 cm−1 shows the nature of the methyl group, and a peak of 1.064 cm^−1^ shows the acetyl group in the polymer.

The reaction that occurs in the bonding agents is caused by the ionization of carboxyl of CA, which is crosslinked with the OH-group on the fiber shown as new bands at 1719–1740 cm^−1^ with the ester group (C=O) stretching mode. This feature peak can be used to assess the effectiveness of the CA crosslinking process on the fiber surface. In untreated fibers, the peak at 1740 cm^−1^ as the carboxylic (C=O) group in lignin, hemicellulose, and pectin can also be associated with the ester group. These compounds are removed by degumming or bleaching treatment by coating the surface of the fiber with nonpolar groups, giving this chemical modification effect a decrease in the hydrophilicity of the fiber.

**Figure 6:**
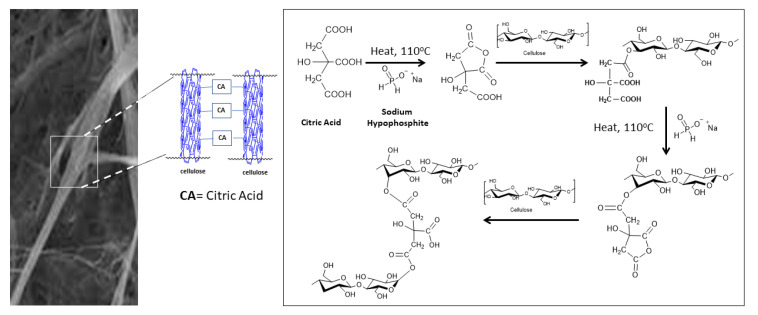
Proposed crosslinking reaction between Cellulose and CA by heating reaction with SHP addition.

The P-bonds of SHP can attach to the fiber when CA dan SHP are heated. A single stretch band appears around the 2375 cm^−1^ area due to the strain reaction from the H-P-H mode originating from the SHP molecules. The peak at 2896 cm^−1^ shows the properties of the methyl or methylene due to C–H stretching. When heated-SHP is applied, the -HC- of the CA change as a result of the loss of carbonyl conjunction C=C. The reaction is due to the ionization of the carboxyl group in the CA, and variation intensity shows C=C at 1623 cm^−1^, which is also in the area fingerprint C–C–C at 1237 cm^−1^. Figure 6 shows the bridging reaction as the reaction of CA with SHP addition. SHP, in this research, played a role as a catalyst.

### 3.4. Surface Morphologies

**Figure 7:**
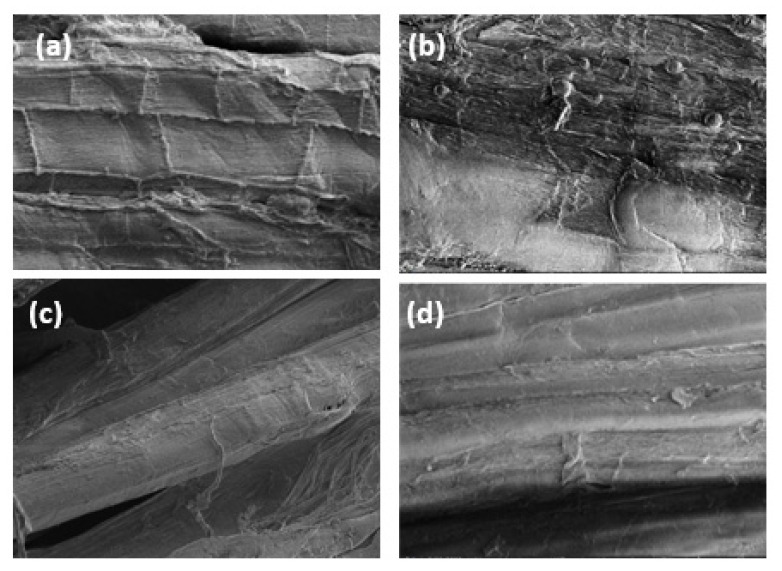
SEM micrograph of the surface of ramie fiber: (**a**) control, (**b**) CA 0%, (**c**) CA 1.875%, and (**d**) CA 1.875% + 1% SHP, 110 °C.

The surface change was analyzed by SEM to confirm the effect of crosslinking CA with ramie (Figure 7). The untreated sample as a control showed a complete tuft structure with a generally flat surface (Figure 7a). According to the hydrophilic cellulose, when the fibers were submerged in water, swelling occurred as a result of water entering the polymer matrix and becoming trapped during submersion, which not significantly altered the mechanical strength (Figure 7b). It can be proved that the addition of CA modified the fiber surface through this crosslinking reaction using the complete ramie fiber structure. Figure 5c shows the effect of CA on the surface change caused by crosslinking between the hydroxyl group of cellulose. The interface became rougher and appeared as vertical segments between the compartment that connected microfibrils in the filament. When the fiber treated with CA/SHP at increased temperature exhibited very fast crosslinking, rendering cellulose highly crosslinked due to its proximity to other molecules (Figure 7d). The surfaces of the fiber look rougher due to the adhesion between microfibrils. This result demonstrated the effectiveness of crosslinking CA with ramie fiber, cell structure denser with regional distribution between cellulose which contributes to the strong tensile strength.

### 3.5. Evaluation of the Formula CA/SHP on Ramie Fiber-Derived Material

The tensile strength of ramie cellulose, including decorticated fiber, degummed-fiber, and ramie yarn, was then examined utilizing the optimal crosslinking CA condition (Figure 7). The chemically and bio-degumming process has the capacity to alter the surfaces of the fiber by hydrolyzing the -OH molecule that is modifying the fiber properties.

**Figure 8:**
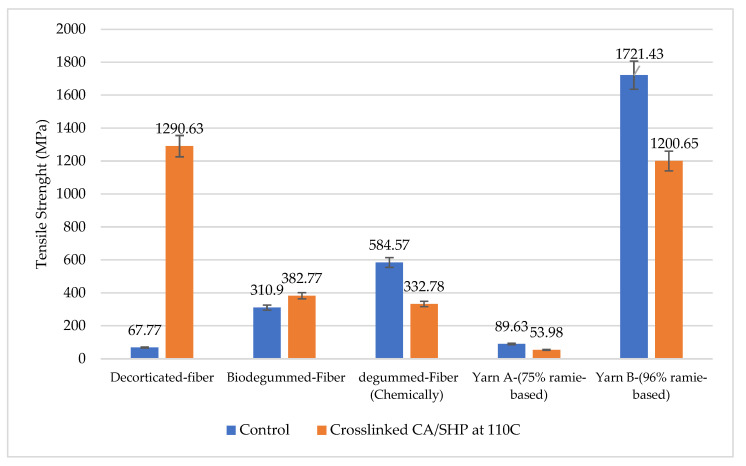
Comparison of the tensile strength due to CA/SHP crosslinking at 110 °C for ramie-based material.

Ramie fiber that went through chemical or biological degumming processing has a fiber strength of 584.54 MPa and 310.90 MPa, respectively. The fiber strength of the bio-degummed-fiber did not significantly increase, which proves that the penetration of the fungal mycelium on the fiber is effective only degrading the lignin and hemicellulose components [34]. The yarn type A and type B, also chemically degummed-fibers, showed a reduction in fiber strength of 39.8%, 30%, and 43.1%, respectively. The treated fibers and yarn exhibited lower mechanical properties when the materials were oriented toward reduced tensile strength. According to these findings, the samples may have less exposure to the -OH bonds on their surface, which could lead to an increase in the number of weak links when CA/SHP is used to react with the material. The tensile strength of the treated fiber and the yarn will gradually deteriorate with high CA concentration with SHP catalyst. These findings demonstrated that the strength of the material is not increased by crosslinking CA/SHP on processed fiber and yarn (Figure 8).

## 4. Discussion

Much work has been completed by many researchers on natural fibers to approach various ways to improve their mechanical properties. Much data have been reported indicating that strengthening the tensile strength of fiber gives varying results due to the possibility of differences in the quality of the initial fiber considering that the natural properties of fibers are very dependent on the location and conditions of the plant and its initial processing. Table 1 shows a comparison of the tensile strength of ramie and other natural materials from different treatments. Several surface treatment methods of ramie can demonstrate the potential strength of the fiber. According to these data, the crosslinking technique using CA and SHP as catalysts has been shown to have a significantly higher tensile strength than the others.

When compared to other natural fibers that have been described, ramie-crosslinked CA/SHP was the strongest fiber among all the treatments that have been reported. Even though there are many other data showing the potential for high strength of natural fibers, due to the lack of details on the procedure and the initial treatment method, we exclude from the discussion.

The interesting thing is an evaluation regarding the ultimate tensile strength of crosslinked CA/SHP with ramie fiber when compared to Built 18Ni300 Maraging Steel is around MPa 1188.6 ± 12.0 MPa–1209.4 ± 16.5 MPa [35]. Martensitic steel is significantly more cost-effective than aluminum. Using optimized design profiles, the steel can deliver high-strength performance that is similar to aluminum and at the same weight as a commercial 1200 MPa grade. This kind of steel has been extensively applied in the automobile industry owing to outstanding mechanical properties: high strength-low weight, high initial work hardening rate, and good formability [36,37]. It has been accepted that fiber strength plays a key role in determining the strength of fiber-reinforced polymer matrix composites. Natural fibers, as light and low-cost materials, when added to a composite mixture, can become a sustainable alternative for other fibers to produce materials with better, harder properties and reduce the weight and density of the final composite [38]. This crosslinking approach provides us with new possibilities for ramie fiber orientation utilization in the form of replacements of synthetic fiber or as functional composites.

In the future, the ramie fiber, which crosslinked with CA in optimum condition, should be recommended for application for Natural Fiber Reinforced Polymer Composite (NFRPC). The bio-composites of NFRPC are categorized into complete and partial green composites [39,40,41,42]. There has been much research completed on some of the significant advancements related to NFRPCs materials that can replace synthetic fibers in numerous applications, including the car manufacturing industry, which has advantages such as being more efficient in terms of production costs, lighter in weight, has an excellent mechanical quality, has biodegradability properties so it is more environmentally friendly, and has economic perspective. By lowering material costs and weight for plastic substitution in the packaging and electrical industries, NFRPC usage offers a great alternative [43]. High-performance fibers could be used for technical textiles, such as for reinforcement of composites in protective garment applications or such as ropes and belts. These characteristics made NFRPCs as special materials for a variety of transportation applications, including airplanes, cars, railroads, and construction [44]. Although there are continuing adverse consequences from its development, in particular, dealing with many challenges associated with the development and application of NFPRC due to the intrinsic qualities of natural fibers (NF) issues related to established quality control. Natural fibers remain far from standardized, nevertheless, and even after going through the same extraction process, individual fibers can exhibit different qualities when compared to one another.

**Table 1:** Comparing tensile strength properties of ramie with different treatments.

No	Material	Treatment	Tensile Strength (MPa)	Reference
1	Ramie Yarn	Polylactic acid (PLA)	38.4–52.7	[45]
2	Ramie Fabric	PLA + Cyclic load treatment	75–85	[46]
3	Ramie fabric	Alkaline + PLA cyclic load	90.9	[45]
4	Composite ramie	diisocyanates	60.1–62.0	[7]
5	Fiber-modified Ramie/Polypropylene (PP)	Amino silicone oil emulsion (ASO)	20.4–28.6	[46]
6	Ramie fiber	Diammonium phosphate (DAP) + Polylactic acid (PLA)	19.6 ± 1.2	[47]
7	Ramie Fiber	Pectinase-DAP/PLA	32.7 ± 1.5	[47]
8	Ramie Fiber	Alkaline-DAP/PLA	33.1 ± 1.6	[47]
9	Ramie Fiber	Silane-DAP/PLA	21.1 ± 1.6	[47]
10	Ramie Fiber	Alkaline-Silane-DAP/PLA	21.5 ± 1.7	[47]
11	Ramie Fiber	Lignin-Based Polyurethane Resin (LPU)	441.19–577.61	[48]
12	Ramie Fiber	Low-viscosity lignin-based polyurethane resin (LPU)	648.7	[10]
13	Ramie Fiber	Tannin-Bio-NIPU	451.3	[9]
14	Ramie Fiber	8% NaOH, a PLA/fiber composite	57.37	[11]
15	Ramie Fiber	Alkaline for prosthetics	62–86	[49]
16	Ramie fiber	Crosslinked Citric acid	172.86	This work
17	Ramie Fiber	Crosslinking Citric Acid + Sodium hypophosphite	1209	This work
18	Banana peel starch	Crosslinking Citric Acid 20–60% (*w*/*w*)	80	[14]
19	Bamboo fiber	Crosslinking Citric acid 5%	0.953–4.202	[50]

The development of ramie fibers that have been impregnated with crosslinking CA/SHP furthermore needs to improve the functionality, especially in terms of the toughness of the material, such as flexural strength, Young’s modulus, density, and color change to be studied as well as fiber structure, dimensions, microfibril and angles, and physical structure, chemical properties, cell dimensions, and interaction of fibers with the matrix must all be taken into account to achieve optimal performance [51,52,53,54] Impurities [55], water absorption [56], orientation [57], and volume fraction [58], which are inherent fiber characteristics, are also important in determining the mechanical properties natural fiber-reinforced polymer composites (NFRPC).

Ramie is being used as a model for developing a national priority in Indonesia as a bioproduct based on textile innovation [59]. This study has significant potential market value if the product prototype created may be oriented towards the completeness of the functional elements of the material according to the needs of the industry, which will be a trend in the future. These exceptional characteristics provide cellulose with special properties such as flame retardance, high swelling ratio, enhanced wrinkle resistance, and antifibrillation that are useful in a variety of applications, including the finishing of textile fibers, biomedical use, and filler applications [32,33,60].

Ramie is used for Multilayer Armor Systems (MAS) and shows the anti-ballistic properties of a composite of three layers of ceramic (wolfram carbide) and 10 layers of ramie nonwoven with epoxy resin as the matrix [61]. The suggestion to use crosslinked CA–ramie produced in this study made it possible to reduce the thickness of the fiber layer used.

## 5. Conclusions

In conclusion, the tensile mechanical properties of ramie fiber with CA/SHP crosslinking have been explored. To obtain the highest tensile mechanical strength that can be obtained as follows:

The addition of CA 0.75–1.875% (*w*/*w*) can show an increase in tensile strength up to 124 MPa–172.86 MPa or about 83–155% compared to the control (67.77 MPa).

- A significant increase in tensile strength was observed more than 19 times when CA/SHP 1% was treated at an activation temperature of 110 °C with a superior tensile strength of 1290.63 MPa. Unfortunately, the effectiveness of CA/SHP crosslinks has not been successfully applied to ramie fibers that have been processed by degumming or that have been spun into yarn.
- The crosslinking ramie with CA/SHP may have occurred, and this has been confirmed by evidence from SEM by a change in the surface structure of the fiber, which is denser as a result of the closeness of the regional distribution between cellulose, which contributes to the strong tensile strength. Besides that, FTIR studies support the evidence of a feature to assess the effectiveness of the CA crosslinking process on the fiber surface. Crosslinking between cellulose and CA in the presence of a reaction between the hydroxyl group of cellulose and the positive carbon of the carboxyl group of CA, which joins to form an ester group which is shown as a new band at 1719–1740 cm^−1^ with the stretching mode of the ester group (C=O).
- The approach described in this study shows the capability of ramie fibers crosslinked with CA to be applied on NFRPC, which can replace synthetic fibers with more sustainable, cost-efficient, stronger, and lighter ones. The NFRPC offers better characteristics for textile functional or aerospace and automotive industries such as cars, rockets, airplanes, railroad, and construction.

## Data Availability

Not applicable.
